# Capsid Structure of Aleutian Mink Disease Virus and Human Parvovirus 4: New Faces in the Parvovirus Family Portrait

**DOI:** 10.3390/v14102219

**Published:** 2022-10-09

**Authors:** Renuk Lakshmanan, Mario Mietzsch, Alberto Jimenez Ybargollin, Paul Chipman, Xiaofeng Fu, Jianming Qiu, Maria Söderlund-Venermo, Robert McKenna

**Affiliations:** 1 Department of Biochemistry and Molecular Biology, Center for Structural Biology, McKnight Brain Institute, College of Medicine, University of Florida, Gainesville, FL 32603, USA; 2 Biological Science Imaging Resource, Department of Biological Sciences, Florida State University, Tallahassee, FL 32306, USA; 3 Department of Microbiology, Molecular Genetics and Immunology, University of Kansas Medical Center, Kansas City, KS 66160, USA; 4 Department of Virology, University of Helsinki, 00014 Helsinki, Finland

**Keywords:** parvovirus, capsid, AMDV, PARV4, cryo-EM, *Amdoparvovirus*, *Tetraparvovirus*, pathogen, VP1u

## Abstract

Parvoviruses are small, single-stranded DNA viruses with non-enveloped capsids. Determining the capsid structures provides a framework for annotating regions important to the viral life cycle. Aleutian mink disease virus (AMDV), a pathogen in minks, and human parvovirus 4 (PARV4), infecting humans, are parvoviruses belonging to the genera *Amdoparvovirus* and *Tetraparvovirus*, respectively. While Aleutian mink disease caused by AMDV is a major threat to mink farming, no clear clinical manifestations have been established following infection with PARV4 in humans. Here, the capsid structures of AMDV and PARV4 were determined via cryo-electron microscopy at 2.37 and 3.12 Å resolutions, respectively. Despite low amino acid sequence identities (10–30%) both viruses share the icosahedral nature of parvovirus capsids, with 60 viral proteins (VPs) assembling the capsid via two-, three-, and five-fold symmetry VP-related interactions, but display major structural variabilities in the surface loops when the capsid structures are superposed onto other parvoviruses. The capsid structures of AMDV and PARV4 will add to current knowledge of the structural platform for parvoviruses and permit future functional annotation of these viruses, which will help in understanding their infection mechanisms at a molecular level for the development of diagnostics and therapeutics.

## 1. Introduction

The *Parvoviridae* is a family of small, non-enveloped, single-stranded DNA viruses [1]. This virus family is divided into three subfamilies: *Parvovirinae*, *Densovirinae*, and *Hamaparvovirinae* [2]. Members of the *Parvovirinae* are further split into ten genera: *Amdo*-, *Arti*-, *Ave*-, *Boca*-, *Copi*-, *Dependo*-, *Erythro*-, *Lori*-, *Proto*-, and *Tetraparvovirus*. The capsids of the *Parvovirinae* are ~260 Å in diameter and their proteins are encoded in the right-hand open reading frame of the viral genomes [3]. Different members of this subfamily either express two or three viral proteins (VPs) that overlap at their C-termini. The larger VPs are extended at their N-termini and have different important functions, including a phospholipase A_2_ (PLA_2_) domain, nuclear localization signals, and/or receptor-binding domains [4,5,6]. These N-terminal-extended VPs are expressed at about a 10-fold lower ratio relative to their shortest (major) VP, in the case of viruses expressing two VPs, 1:10 (VP1:VP2), and in the case of the viruses expressing three VPs, 1:1:10 (VP1:VP2:VP3) [7]. Their icosahedral capsids are composed of 60 VPs that assemble via two-, three-, and five-fold symmetry-related VP interactions. The viral capsids are critical components of the viral infectious life cycle: they protect the viral genome, are the determinants of the tissue type and host that is targeted (tropism), and remain intact while the virus escapes the endo-/lysosomal pathway and trafficks to the nucleus where the genome is released [8], leading to viral genome replication.

The capsid structures of several members of the *Parvovirinae* have been determined by X-ray crystallography and/or cryo-electron microscopy (cryo-EM) [3,9,10,11]. However, to date, these capsid structures originate from only four of the ten genera (*Boca*-, *Dependo*-, *Erythro*-, and *Protoparvovirus*). In all these structures, the N-terminal extended regions of the larger VPs, as well as the N-terminal 20–40 amino acids (aa) of the smaller VPs, have not been resolved [3]. They are believed to be highly flexible, likely due to a glycine-rich region near the N-terminus of the major VP, and are located in the interior of the capsid. Upon receptor-mediated endocytosis and subsequent acidification of the endosome, the VP1 unique (VP1u) with its PLA_2_ enzymatic domain is externalized [12]. One described exception is parvovirus B19 (B19), of the *Erythroparvovirus* genus, which possesses a receptor-binding domain in its VP1u which needs to be located on the exterior side of the capsid for attachment to its target cells [6]. The structurally ordered regions of the VPs, which comprise most of the major VPs, display significant similarities among the members of the different genera, despite low sequence identities (10–30%) [13]. All VP monomers of members in the *Parvovirinae* consist of a core, eight-stranded (βB to βI), anti-parallel β-barrel motif, also known as a jelly-roll motif, with a BIDG sheet that forms the inner surface of the capsid. Additionally, a βA strand that runs anti-parallel to the βB strand and a helix αA that is located between the strands βC and βD are also conserved [3]. Between the β-strands of the VPs, connecting loops are inserted, which form the surface of the capsid. These loops are named after the connecting β-strands; for example, the HI loop connects the βH and βI strands. These surface loops exhibit the greatest amino acid sequence and structural diversity among members of the same genus and between the different genera. Differences at the apexes of these loops are termed variable regions (VRs), defined as two or more amino acids with Cα positions greater than 2 Å apart when their VPs are superposed. Despite these structural differences on the capsid surface, members of the *Parvovirinae* share the same overall characteristic features, including channels at the icosahedral five-fold symmetry axes, protrusions at or around the three-fold symmetry axes, and depressions at the two-fold symmetry axes and surrounding the five-fold channels, which depressions are separated by a raised region termed the two/five-fold wall [3].

Members of the *Parvovirinae* infect a wide range of vertebrate hosts, including humans [1,14]. Parvovirus 4 (PARV4) is a human parvovirus, first reported in 2005 in the serum of an intravenous drug user infected with hepatitis B virus [15]. The virus has been detected worldwide in plasma and different tissues, and it seems to be transmitted parenterally to hemophiliacs and injection-drug users, but with no clear clinical manifestations (reviewed in [14]). However, a recent study reported a strong association between PARV4 and individuals showing severe respiratory illness, and its DNA was found in cerebrospinal fluid from two children with encephalitis [16,17]. PARV4 has been assigned to the genus *Tetraparvovirus*, along with other members infecting pigs, sheep, cattle, yaks, and bats [18].

Aleutian mink disease virus (AMDV) is a member of the genus *Amdoparvovirus*. Viruses of this genus primarily infect carnivores, as well as some rodents and bats [19]. AMDV is known to cause Aleutian disease, which results in the enlargement of the kidneys, the spleen, and the lymph nodes and manifests in deadly plasmacytosis and hyperglobulinemia [20]. Outbreaks of this virus are a significant threat to mink farms. Currently, there are no vaccines available against AMDV [20].

To date, for PARV4, AMDV, and other viruses in their genera, no capsid structures have been determined. This study reports the capsid structures of PARV4 and AMDV determined by cryo-EM at 3.12 and 2.37 Å resolutions, respectively. These capsid structures of members of the genera *Tetraparvovirus* and *Amdoparvovirus* have been compared with capsid structures of viruses of the known genera—the *Boca*-, *Dependo*-, *Erythro*-, and *Protoparvovirus* capsids. Despite low sequence identities, these viruses share common capsid features with the viruses in the *Parvovirinae* subfamily but display major differences in the surface loops, with several large insertions and/or deletions. In particular, the capsid of AMDV possesses the largest major VP, with ~60 to 115 additional aa compared to other *Parvovirinae* members for which capsid structures have been determined. In contrast, AMDV’s VP1u is very short and does not contain a PLA_2_ domain. On the other hand, PARV4 possesses a VP1u that is much longer than those of other members of the *Parvovirinae*. It contains a PLA_2_ domain and likely also a receptor-binding domain.

These studies provide a structural platform for functional annotation of these viruses that will help to understand their disease mechanisms at a molecular level. This information could be applicable in the development of therapeutics.

## 2. Materials and Methods

### 2.1. Production of Virus-like Particles (VLPs)

The open reading frames expressing VP2 for PARV4 (nt: 3464–5122; accession no.: AY622943) and AMDV (nt: 2406–4349; accession no.: M20036) were cloned into the pFastBac1 plasmid vector. For the AMDV construct expressing VP1 and VP2, nt 2204–4349 were cloned into pFastBac1, excluding the intron sequences (nt 2214–2286), and the VP1 start codon was changed to ACG. These constructs were used to generate the recombinant baculoviruses by following the standard Bac-to-Bac Baculovirus Expression System protocol (Invitrogen, Waltham, MA, USA). The recombinant baculoviral stocks were used to infect Sf9 cells in the mid-logarithmic phase at a multiplicity of infection (MOI) of 5 plaque-forming units (PFUs). The Sf9 cells were harvested after 72 h by centrifugation at 1000× *g* for 20 min at 4 °C. Following centrifugation, the supernatants were separated from the cell pellets. The Sf9 cell pellets were resuspended in TNTM buffer (25 mM Tris-HCl, 100 mM NaCl, 0.2% Triton X-100, 2 mM MgCl_2_, pH 8.0), and the supernatants were subjected to polyethylene glycol (PEG) treatment with the addition of 10% (*w*/*v*) PEG 8000 and overnight stirring at 4 °C. The PEG-treated samples were centrifuged at 14,300× *g* for 90 min at 4 °C, followed by resuspension of the PEG pellets in TNTM buffer. Furthermore, the resuspended PEG and cell pellets were combined and lysed using the LM10 Microfluidizer (Microfluidics, Westwood, MA, USA) at 5000 psi. The lysates were benzonase-treated and clarified by centrifugation at 12,000× *g* for 30 min to remove cell debris. The supernatants were loaded onto a 20% sucrose cushion (*w*/*v* sucrose in TNTM buffer) and centrifuged at 45,000 rpm, using a Ti70 rotor for 3 h at 4 °C. The sucrose cushion pellet was resuspended in TNTM buffer, loaded onto a 10–40% sucrose step gradient (*w*/*v* sucrose in TNTM buffer), and centrifuged using a SW41 rotor for 3 h at 4 °C. The sample fractions were then recovered by fractionation, analyzed by SDS-PAGE, dialyzed in phosphate-buffered saline (PBS), concentrated to ~1 mg/mL using Apollo concentrators (Orbital Biosciences, Topsfield, MA, USA), and stored at −20 °C.

### 2.2. Cryo-EM Data Collection

Purified VLPs were loaded onto a glow-discharged holey carbon-coated grid (Quantifoil, Großlöbichau, Germany) and incubated at 4 °C at 95% humidity for 30 s in a Vitrobot Mark IV (Thermo Fisher Scientific, Waltham, MA, USA). Following this, excess sample was blotted by the machine and the grids were vitrified by plunging into liquid ethane. The ice quality of the vitrified grids as well as sample distribution were probed by imaging on a 200 kV FEI Tecnai G2 F20-TWIN transmission electron microscope (FEI, Hillsboro, OR, USA) at ~20 e^−^/Å^2^. After confirming the quality of the grid, high-resolution data collections were performed at the Biological Science Imaging Resource (BSIR) at Florida State University. Data were collected on a Titan Krios electron microscope operating at 300 kV and equipped with the DE64 or DE-Apollo direct electron detector (Direct Electron, San Diego, CA, USA), with 52 or 102 movie frames collected per micrograph at a total electron dose of ~60 e^−^/Å^2^. Furthermore, motion-corrected micrographs were obtained by aligning the movie frames, using MotionCor2 (version 1.5.0) with dose weighting [21].

### 2.3. 3D Image Reconstruction

The motion-corrected micrographs were imported into the cisTEM software package, which was used for 3D image reconstruction of the PARV4 and AMDV capsids [22]. CTF estimation was used to exclude micrographs of poor quality, as well as micrographs with overlapping or damaged particles. Particles were picked automatically and subjected to 2D classification by imposing icosahedral symmetry. The 2D classes containing clear virus features were used for generation of an ab initio model by 3D reconstruction. The 3D model was then auto-refined using default settings, and the final density map was sharpened with a pre-cutoff B-factor value of −90 Å^2^ and a post-cutoff B-factor value of 20 Å^2^. The resolution of the density map was calculated based on a Fourier shell correlation of 0.143 (Table 1).

### 2.4. Model Building

In silico models of PARV4 and AMDV were generated using the SWISS-MODEL homology-modeling server [23]. Furthermore, VIPERdb was used to generate a T = 1 icosahedral capsid from the in silico model [24]. The newly generated capsid 60-mer was then docked into the EM density map using the ‘fit in map’ feature of UCSF Chimera [25]. The fit of the 60-mer with respect to the EM density map was then improved by adjusting the voxel size, which maximizes the correlation coefficient. The voxel-size-adjusted EM density map was then imported into Coot, where manual model building and real-space refinement tools were used to improve the 60-mer model [26]. Finally, the 60-mer was automatically refined using the real-space-refine subroutine in PHENIX, which also provided refinement statistics [27]. Based on their primary amino acid sequences, in silico models of VP1u and AMDV VR-VII were generated using AlphaFold v2.0 run on HiPerGator (UF research computing). 

## 3. Results and Discussion

### 3.1. Determination of the Human Parvovirus 4 Capsid Structure 

**Figure 1:**
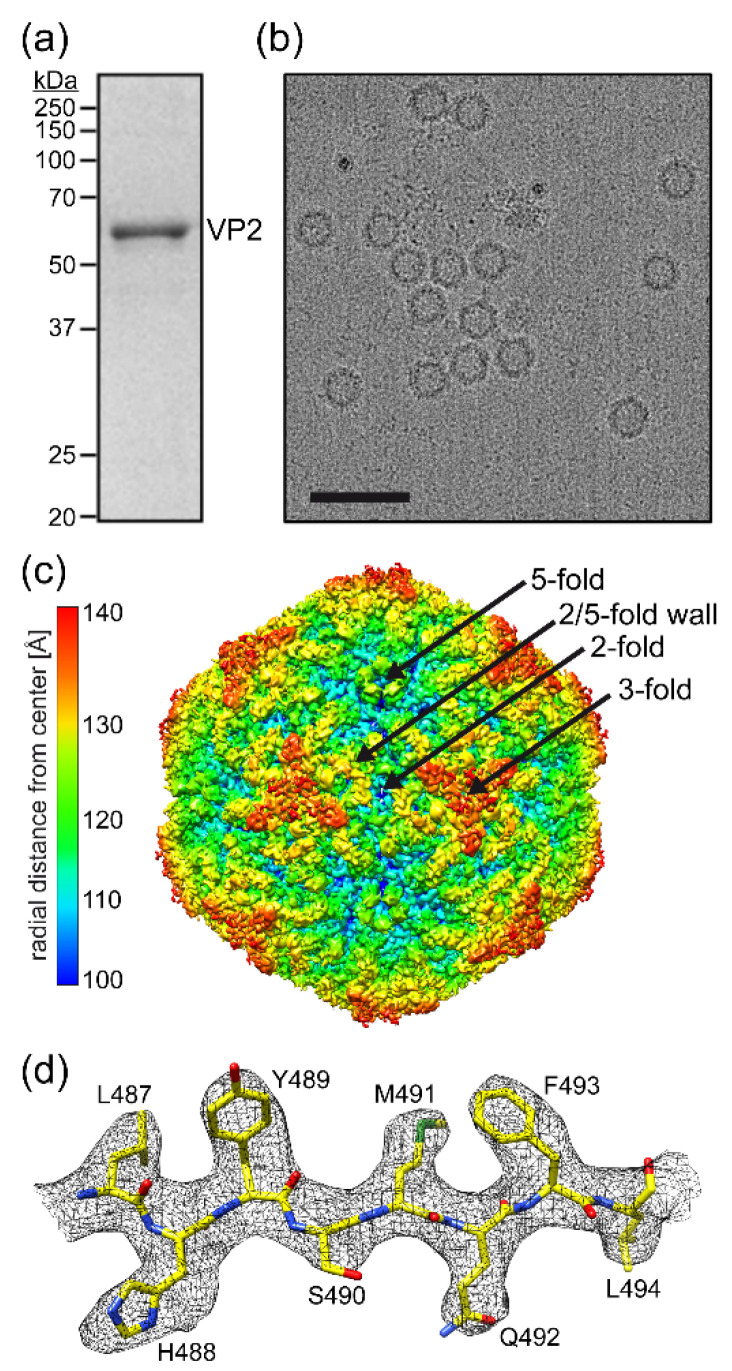
Determination of the PARV4 capsid structure. (**a**) SDS-PAGE of purified PARV4 VLPs, with a band at ~60 kDa equivalent to the size of VP2. (**b**) Example cryo-electron micrograph of PARV4. Scale bar: 500 Å. (**c**) The capsid surface density map contoured at a sigma (σ) threshold level of 1.0. The map is radially colored (blue to red) according to distance to the capsid center, as indicated by the scale bar on the left. The approximate icosahedral two-, three-, and five-fold axes are indicated. (**d**) Amino acid residues modeled for the βI strand are shown inside their density maps (in black). The amino acid residues are as labeled and shown as stick representations and colored according to atom type: C = yellow, O = red, N = blue, S = green. Panels (**c**,**d**) were generated using UCSF-Chimera [25].

Following the production and purification of PARV4-virus-like particles (VLPs), the purified fraction, analyzed by SDS-PAGE, contained a band consistent with PARV4 VP2, migrating at ~60 kDa (Figure 1a). Cryo-EM micrographs showed intact particles ~250 Å in diameter (Figure 1b). Thus, the PARV4 sample was deemed suitable for structural determination by cryo-EM. Three-dimensional-image reconstruction of the PARV4 capsids, utilizing a total of 5248 individual particles, resulted in a resolution of 3.12 Å, based on an FSC threshold of 0.143 (Table 1). The reconstructed capsid of PARV4, the first structure of the genus *Tetraparvovirus*, displayed familiar surface features, similar to those observed in the capsids of other genera in the *Parvovirinae* subfamily, with channels at the icosahedral five-fold axes, protrusions near the three-fold axes, and depressions at the two-fold axes and surrounding the five-fold axes (Figure 1c) [3]. Despite these common features, the specific shapes of the protrusions and depressions were found to be unique when compared to other parvoviruses. Overall, the PARV4 capsid has a “flatter” appearance, similar to some densoviruses [3], since the three-fold protrusions do not project radially outwards and instead are positioned towards the two-fold, three-fold, and five-fold symmetry axes. This results in very small depressions at the two-fold axes and more segmented depressions surrounding the five-fold channel. While most viruses of the *Parvovirinae* subfamily possess three separated protrusions surrounding the three-fold symmetry axis [3], in PARV4, density near the three-fold axis fuses the three-fold protrusions in the shape of a concaved triangle. Additionally, fused three-fold protrusions have been previously observed for various animal protoparvoviruses [13], though with a differently shaped three-fold region compared to PARV4. Structural order in the density map of the PARV4 VP started at lysine 15 (VP2 numbering). The amino acids in the VP monomer were generally well-ordered (Figure 1d) to the C-terminal leucine 552, with the exception of aa 380–401, located near the three-fold symmetry axis, where only diffuse electron density was observed at a sigma (σ) threshold of ~1, preventing reliable placement of the amino acid chain.

### 3.2. Determination of the Aleutian Mink Disease Virus Capsid Structure

**Figure 2:**
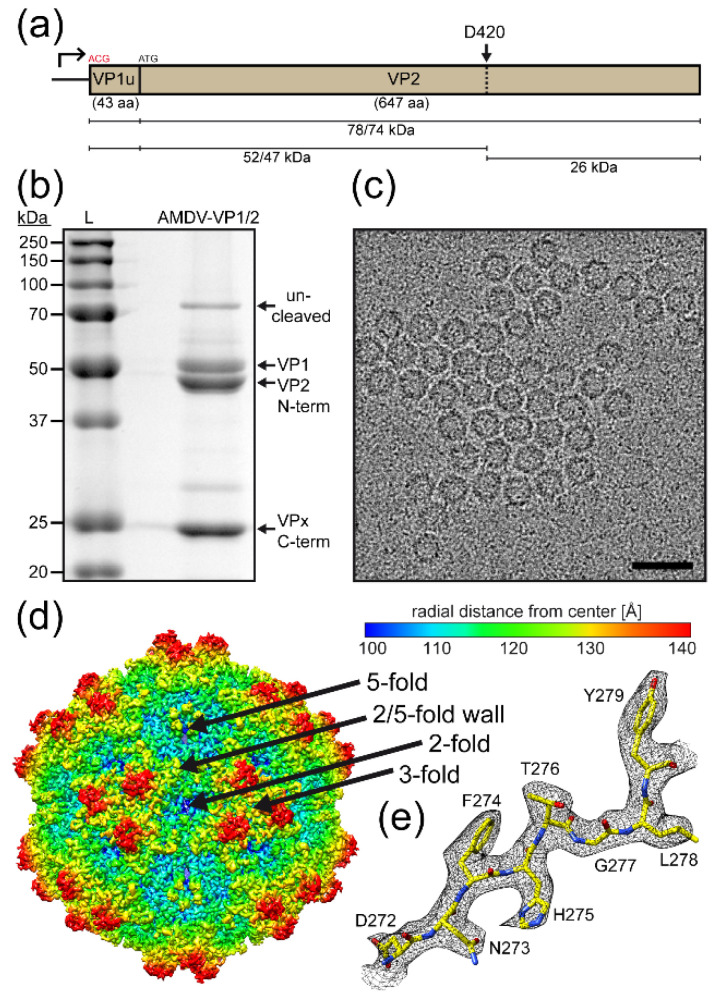
Determination of the AMDV capsid structure. (**a**) Depiction of the AMDV-VP1/2 expression construct. A cleavage of the AMDV VPs at D420 has been described previously [28]; the results for the VP1 and VP2 fragments as shown below. (**b**) SDS-PAGE of purified AMDV VLPs with bands at ~75, 52, 47, and 25 kDa equivalent to the size of uncleaved VP2, the N-terminal VP1 and VP2 fragments, and the C-terminal VPx fragment, respectively. (**c**) Example cryo-electron micrograph of AMDV. Scale bar: 500 Å. (**d**) The capsid surface density map contoured at a sigma (σ) threshold level of 2.0. The map is radially colored (blue to red) according to distance to the capsid center, as indicated by the scale bar on the right. The approximate icosahedral two-, three-, and five-fold axes are indicated. (**e**) Amino acid residues modeled for the βG strand are shown inside their density maps (in black). The amino acid residues are as labeled and shown as stick representations and colored according to atom type: C = yellow, O = red, N = blue. Panels (**d**,**e**) were generated using UCSF-Chimera [25].

Similar to PARV4, AMDV VLPs were produced using the Bac-to-Bac system. However, in contrast to PARV4 VLPs, the AMDV VLPs composed of VP1 and VP2 were generated by modifying the VP1 start codon to ACG (Figure 2a). The same strategy was not possible for PARV4, as its VP1u region is very long and contains multiple ATGs prior to the VP2 start codon. For AMDV, the VP1u region is very short (43 aa) compared to those of other members of the *Parvovirinae* and does not possess a PLA_2_ domain. In addition, AMDV capsid proteins were shown to be cleaved by caspases in Crandell feline kidney (CrFK) cells [28]. This cleavage event was also observed following the production and purification of AMDV VLPs in insect Sf9 cells (Figure 2b). Nonetheless, this cleavage event did not affect the integrity of the capsids, as homogenous, intact particles ~250 Å in diameter were visible by cryo-EM (Figure 2c). Previously, pan-caspase inhibitors were shown to be effective in preventing this cleavage in CrFK cells [28] however, this inhibitor was not functional in Sf9 cells.

As the capsids appeared uniform, the AMDV sample was deemed suitable for high-resolution structural determination. Three-dimensional-image reconstruction of a total of 93,393 individual AMDV capsids resulted in a resolution of 2.37 Å, based on an FSC threshold of 0.143 (Table 1). The reconstructed capsid of AMDV, the first determined structure of the genus *Amdoparvovirus*, also displayed familiar surface features, as described above, with channels at the icosahedral five-fold axes, protrusions near the three-fold axes, and depressions at the two-fold axes and surrounding the five-fold axes (Figure 2d) [3]. Previously, the capsid structure of AMDV was determined by cryo-EM at ~22 Å resolution and showed the same overall capsid surface features [29]. 

Unlike PARV4, the three-fold protrusions were separated and radiated further outwards (the maximum capsid diameter of AMDV being ~300 Å compared to ~270 Å for PARV4). Overall, the AMDV capsid looks similar to bufavirus capsids [13]. Structural ordering in the density map was observed, starting at threonine 42 (VP2 numbering). The amino acids, including their side chains in the VP monomers, were generally very well-ordered (Figure 2e) to the C-terminal tyrosine 647, with the exception of the apexes of the loops at the three-fold protrusion, involving aa 93–94 and 238–239, which showed only weak density at a sigma (σ) threshold of ~1, preventing reliable placement of the amino acid side chains. In addition, no density was observed for aa 420–449. This stretch of amino acids is located at the caspase cleavage site (Figure 2a). 

In addition to the AMDVs containing VP1 and VP2, the capsid structure of AMDV VLPs only containing VP2 was also determined by cryo-EM at 3.1 Å resolution (data deposited in EMDB). However, the capsid structure was indistinguishable from the VP1/VP2 capsids and similar to other parvovirus capsid structures, due to the low copy numbers of the minor VPs and the flexibility of their N termini [3].

### 3.3. The Three-Fold Protrusions Are Formed by Different Loops in PARV4 and AMDV

**Figure 3:**
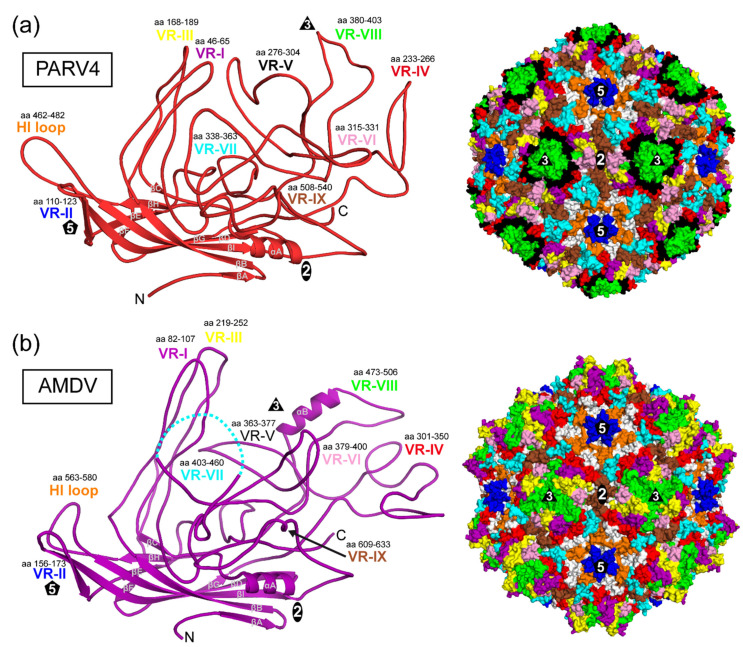
The PARV4 and AMDV VP structures. (**a**) Ribbon diagram (left) of PARV4 VP. The conserved β-barrel core motif (βB–βI), βA, the αA helix, the N- and C-termini are indicated. The VRs (VR-I to -IX) are labeled and the approximate icosahedral two-, three-, and five-fold axes are represented by ovals, triangles, and pentagons, respectively. Right: The location of the VRs, colored as in the ribbon diagram, on the capsid surface of PARV4. (**b**) Ribbon diagram (left) and capsid surface map of AMDV VP, as in (**a**).

The VP monomers of PARV4 and AMDV (Figure 3a,b) conserve the β-barrel core motif (βB–βI) with the additional βA strand and the α-helix A (αA) observed in other parvoviruses. The structurally ordered N-terminus is situated in both viruses in approximately the same location underneath the βBIDG sheet. However, for most capsid structures of the *Parvovirinae*, the N-terminus is located directly below the five-fold channel, except for parvovirus B19 [3]. This is mediated by a conserved hydrophobic valine to leucine interaction of the N-terminus and the DE loop (canine parvovirus (CPV): V38-L169, human bocavirus 1 (HBoV1): V36-L151, adeno-associated virus serotype 2 (AAV2): V221-L336) [30,31]. Neither parvovirus B19 nor PARV4 conserves these residues, which might explain their differently structured VP N-termini. In contrast, AMDV conserves both residues, V38 and L175, but valine 38 is embedded in a 17 aa-long glycine-rich sequence element (aa 22–39) which introduces a lot of flexibility and might prevent interaction with the DE loop.

Large loops are inserted between the β-strands that form the surface of the capsid (Figure 3). The largest loop is the GH loop with multiple subloops comparable to other capsid structures of members in the *Parvovirinae*. Despite the significant structural variability of these surface loops, the base of each loop originates from approximately the same location in the capsid core as for all viruses of this subfamily. Thus, the previously described variable-region (VR) terminology for other genera can also be applied to PARV4 and AMDV to describe these loops. 

The PARV4 capsid utilizes VR-II to form the five-fold channel; VR-I, VR-III, and VR-IX for the two-/five-fold wall; and VR-IV, VR-V, and VR-VIII for the three-fold protrusions (Figure 3a). The fused three-fold protrusions are caused by VR-VIII with its subloop leading towards the three-fold symmetry axis. Interestingly, the fused three-fold protrusions of the animal protoparvoviruses (e.g., CPV) are generated by the equivalent loop, though without any sequence similarity [10]. The small depression at the two-fold symmetry axis is the result of VR-VI covering most of the depression from both sides (Figure 3a, right panel). Around the five-fold channel, the depression is interrupted by VR-VII interacting with the HI loop.

**Figure 4:**
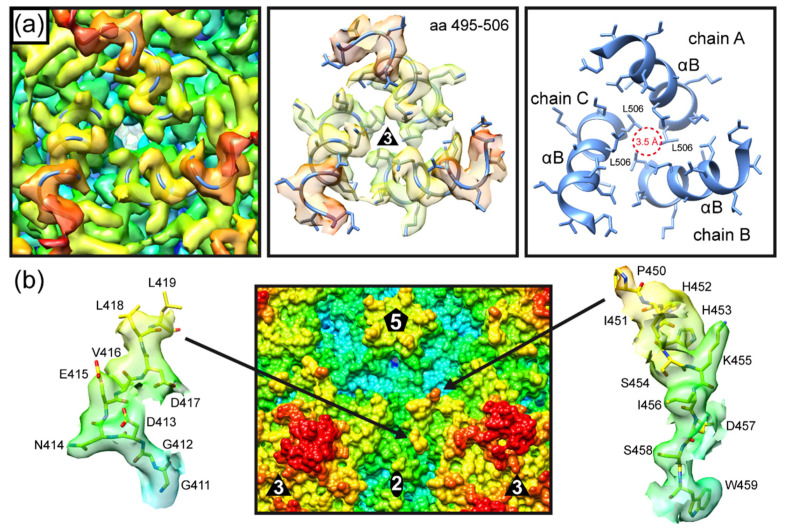
Unique capsid features of AMDV. (**a**) The α-helices B of three symmetry-related monomers are positioned around the three-fold symmetry axis. Left and center panel: the ribbon diagram of aa 495–506 fitted into the density map of AMDV (center panel with amino acid side chains). Right panel: The ribbon diagrams are shown without the map. Leucine 506 is located near the center, surrounding a pore approximately 3.5 Å in diameter. (**b**) AMDV’s cleaved VR-VII is located at the two/five-fold wall. The N-terminal end (leucine 419), shown inside the density map (left), is located closer to the two-fold symmetry axis, while the C-terminal arm of the loop (right model inside the density map) is located closer to the five-fold symmetry axis. No density was observed for aa 420 to 449.

The AMDV capsid also utilizes VR-II to form the five-fold channel (Figure 3b). However, in contrast to PARV4, the elongated VR-I and VR-III (15 and 18 aa longer, respectively) are responsible for the three-fold protrusions in AMDV. The region at the three-fold symmetry axis between the protrusions is primarily formed by VR-VIII, while VR-V is not surface-exposed. The VR-VIII loop possesses an additional α-helix B (Figure 3b). To date, only the viruses of the *Bocaparvovirus* genus have been described as possessing a long α-helix at the capsid surface [11,32]. However, in contrast to AMDV, the surface helix of the bocaviruses is located in VR-III at the two/five-fold wall. The α-helix B in AMDV is located very close to the three-fold symmetry axis. In fact, three helices from three symmetry-related VPs are positioned around the three-fold symmetry axis, creating a small pore with a diameter of ~3.5 Å (Figure 4a). Interestingly, leucine 506 is located at the center and represents the narrowest point of the three-fold pore. In the five-fold pore with a larger diameter (~7.6 Å), leucines (L175) are also located at its narrowest point. It remains to be seen whether the three-fold pore fulfills specific functions in the viral life cycle. Possible amino acid substitutions of leucine 506 may help to characterize the pore, as in experiments performed on the five-fold channel [30,33].

The two/five-fold wall of the AMDV capsid is formed by VR-IV, VR-VII, and VR-IX (Figure 3b). However, due to the cleavage of the AMDV VPs at D420, no density was observed for aa 420 to 449, but both ends of VR-VII, leucine 419 and proline 450, were visible at the capsid surface (Figure 3b and Figure 4b). If the missing 30 aa were to form a loop, it would radiate further outwards than the three-fold protrusions (VR-I and VR-III). However, given that two unrelated cell lines, feline cells [28] and insect cells, resulted in the same cleavage event, it may be that the DLLD/G caspase cleavage site [34] is cut in minks as well. Thus, VR-VII might have a flexible, linear amino acid element on the capsid surface. AlphaFold 2 predictions of the 30 aa stretch suggest a helix-turn-helix motif for this sequence [35]. More research to determine the importance of the VR-VII sequence, including the potential helix-turn-helix for the viral life cycle, is needed.

### 3.4. Inter-Genus Sequence and Structural Comparisons

**Figure 5:**
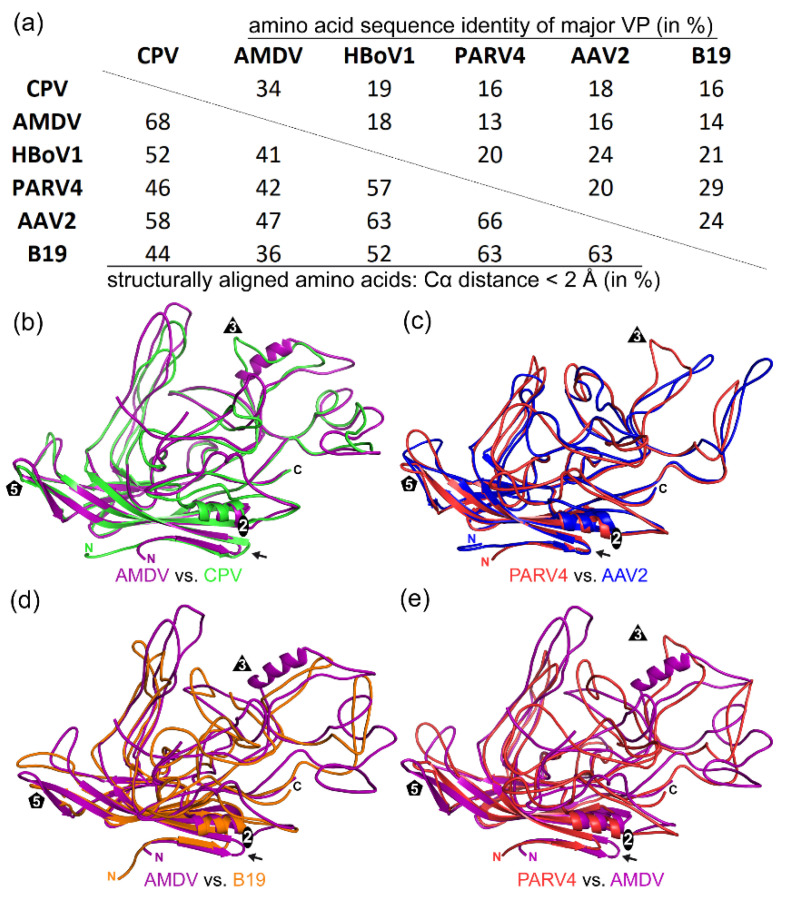
Sequence and structural comparison. (**a**) Amino acid sequence identity comparison of the major VPs (top right) between CPV, AMDV, HBoV1, PARV4, AAV2, and B19 (in %). Structural similarity (bottom left) was defined as the percentage of aligned Cα atoms of the amino acid chain within 2 Å distance when the capsid structures were superposed. (**b**) Superposition of AMDV (purple) and CPV (green). The N- and C-termini and the approximate icosahedral two-, three-, and five-fold axes are indicated. (**c**) Superposition of PARV4 (red) and AAV2 (blue). (**d**) Superposition of AMDV (purple) and B19 (orange). (**e**) Superposition of PARV4 (red) and AMDV (purple). Arrows indicate the position of the AB loop.

For capsid sequence and structural comparison of PARV4 and AMDV with the subfamily *Parvovirinae*, representative members of the genera were chosen for which structural data were available. At the time of this study, capsid structures were available for four of the ten *Parvovirinae* genera. The representative members that were selected for the genera members used were as follows: for the *Bocaparvovirus* genus, HBoV1; for the *Dependoparvovirus* genus, AAV2; for the *Erythroparvovirus* genus, B19; and for the *Protoparvovirus* genus, CPV. Overall, the capsid amino acid sequence identities of viruses from different genera were low, irrespective of the genera compared. They ranged from 13% (AMDV vs. PARV4) to 34% (AMDV vs. CPV) (Figure 5a). In a previous study characterizing distant members of the *Protoparvovirus* genus, even intra-genus capsid sequence identity was as low as 30% [10]. Thus, AMDV showed a close relationship to the *Protoparvovirus* genus (34% to CPV) and PARV4 showed a close relationship to the *Erythroparvovirus* genus (29% to B19). This also translated to a high structural similarity of 68% for the AMDV capsid structure aligned with the capsid structure of CPV (Figure 5a,b). 

Common features between the AMDV and CPV capsids include the three-fold protrusions formed by VR-I and VR-III, VR-V not being surface-exposed, and the extended AB loop on the inside of the capsid, which has been shown to be involved in nucleotide binding for the protoparvoviruses [36]. For PARV4 and B19, the 29% sequence identity translated to 63% structural similarity (Figure 5a). However, despite only having a sequence identity of 20%, the AAV2 capsid had the highest structural similarity to the PARV4 capsid (Figure 5c). Common features between the PARV4 and AAV2 capsids include the three-fold protrusions formed by VR-IV, VR-V, and VR-VIII and the two-/five-fold wall formed by VR-I, VR-III, and VR-IX [3]. Furthermore, while the capsids of PARV4 and CPV are not very similar, both viruses possess a similar subloop in VR-VIII leading towards the three-fold symmetry axis, which results in the fused appearance of the three-fold protrusions (compare the green ribbon in Figure 5b with the red ribbon in Figure 5c). 

The AMDV capsid showed the lowest structural similarity to the B19 capsid, with 36% structurally aligned residues (Figure 5a,d), and the PARV4 capsid showed the lowest structural similarity to the AMDV capsid, with 42% structural similarity (Figure 5a,e). In these structural comparisons, the only region that aligned was the core jelly-roll motif. 

### 3.5. Unusual Length of AMDV VP1u, VP2, and VR-VII

**Figure 6:**
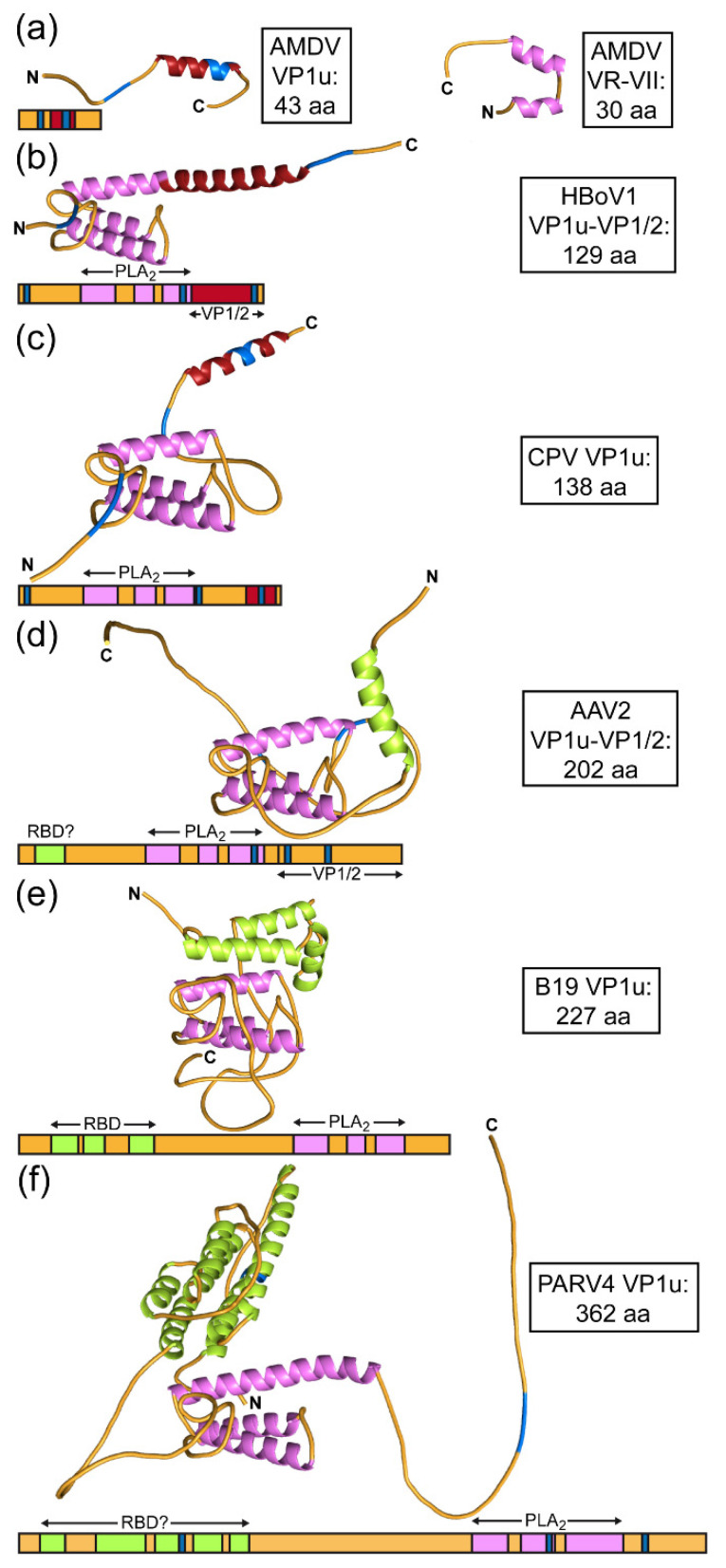
Structural prediction for VP1u. Alphafold [35] predictions utilizing the primary sequences of (**a**) the AMDV VP1u and VR-VII regions, (**b**) the HBoV1 VP1u and VP1/2 common region, (**c**) the CPV VP1u, (**d**) the AAV2 VP1u and VP1/2 common region, (**e**) the B19 VP1u, and (**f**) the PARV4 VP1u. The helices of the PLA2 domains are colored magenta, the helices of (potential) receptor-binding domains (RBD) light green, the C-terminal helices dark red, and the basic regions blue. The N- and C-termini are indicated.

Members of the genus *Amdoparvovirus* (and *Aveparvovirus*) differ from most viruses in the subfamily in that they do not possess the common PLA_2_ sequence motif in the VP1u region (Figure 6a). Furthermore, their VP1u is short (only 43 aa) in comparison to other viruses of the *Parvovirinae* (Table 2). For most viruses, the VP1u region is ~100–200 amino acids longer. In the case of PARV4 the VP1 open reading frame codes for a 362 aa VP1u region. However, the exact start codon for VP1 is still debated [14,37]. In contrast to AMDV’s short VP1u, its major capsid protein is among the largest in the subfamily. Compared with other members of the *Parvovirinae*, it displays multiple significant insertions and some deletions, resulting in a ~60 to 120 aa larger VP2 (Table 2). At the N-terminus it possesses the longest glycine-rich region of the family [3]. However, most insertions are located at the capsid surface. In fact, AMDV has the longest loop for VR-I, VR-III, VR-IV, and VR-VII among natural *Parvovirinae* isolates for which structures have been determined (Table 2). The only major deletion is located in VR-V, which is shared with CPV and other protoparvoviruses, as this loop is not surface-exposed in these viruses [3]. Given the differences in the lengths of VP1u and VP2 relative to other viruses, it may be possible that some functions of the VP1u region are conducted by the major capsid protein. One such region that may exhibit additional functions could be VR-VII. This loop in AMDV is 31–42 aa longer than in any other genus (Table 2). Furthermore, one side of the loop is cleaved directly at the capsid surface (Figure 4b), which may allow the remaining loop to fold according to its function. The sequence in this region is specific to the genus *Amdoparvovirus*, and structural predictions suggest a helix-turn-helix motif (Figure 6b). 

Helix-turn-helix motifs are predicted to be present in the PLA_2_ domains of the VP1u for most viruses of the *Parvovirinae* (Figure 6). Despite low amino acid sequence identities for the PLA_2_ domains ranging from 30–50%, the predicted α-helices of the different viruses are well superposable. In addition to the PLA_2_ domain, some viruses may have other functional domains in their VP1u and/or VP1/2 common regions. Both HBoV1 and CPV possess α-helices near their C-termini (Figure 6b,c). CPV’s α-helix in this region has high sequence similarity to the only α-helix in AMDV VP1u (Figure 6a). AAV2, B19, and PARV4 display α-helices near the N-terminus of VP1u (Figure 6d–f). For Parvovirus B19, the presence of a receptor-binding domain at the N terminus, which is predominantly α-helical, has been described before [6,38]. A recent study showed that AAV2 and other AAV serotypes are dependent on a G protein-coupled receptor, GPR108, for effective transduction and that the interaction is dictated by the VP1u region [39]. While the exact amino acids in VP1u for the interaction have not been determined, it is possible that the N-terminal region including the α-helix (aa 9–24) are involved (Figure 6d). PARV4, with its very long VP1u region, displays a substantial α-helical region near the N-terminus. Five α-helices (aa 15–121) were predicted, which could represent a receptor-binding domain (Figure 6f). To date, no receptor has been identified for PARV4. Due to the size, it is highly likely that most, if not all, of the VP1u region of PARV4 is localized on the exterior side of the capsid, similar to that of Parvovirus B19 [40], which would enable PARV4 to bind its receptor with its potential VP1u RBD.

## 4. Conclusions

**Figure 7:**
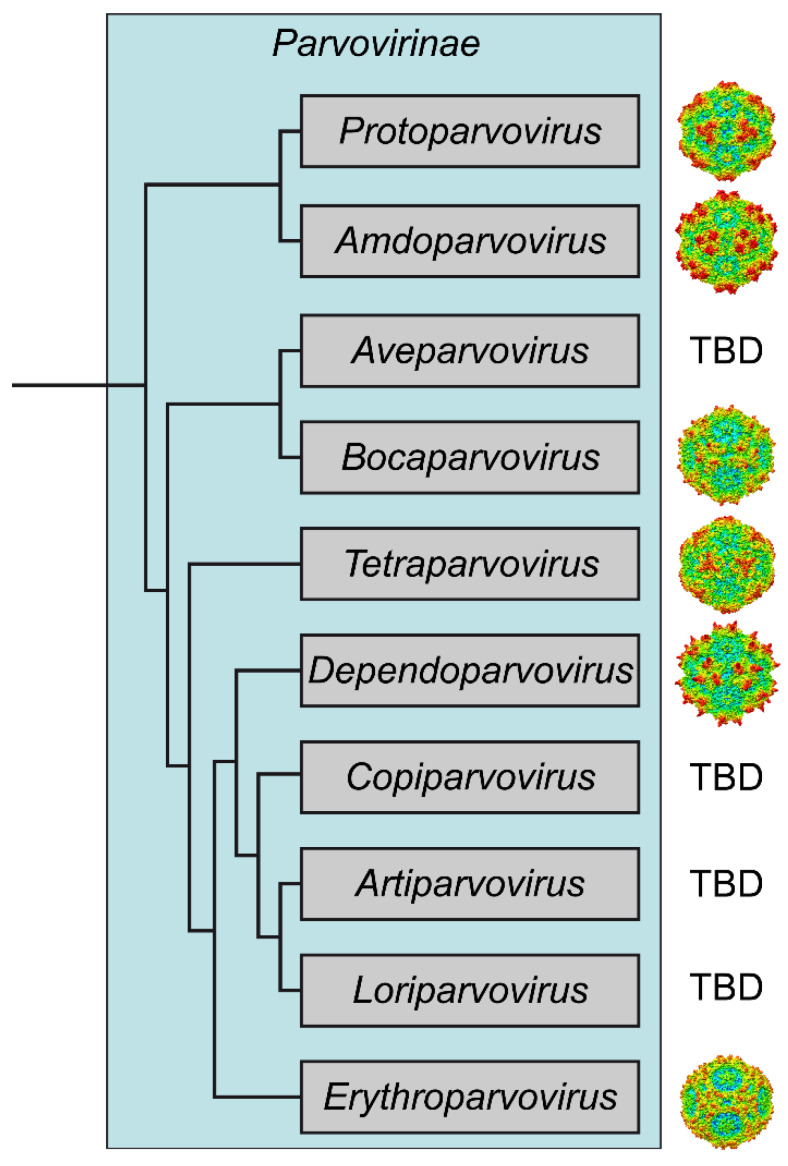
Cladogram of the *Parvovirinae* subfamily based on Penzes et al. [2]. The representative members of the genera for which capsid structures have been determined are CPV (*Protoparvovirus*), AMDV (*Amdoparvovirus*), HBoV1 (*Bocaparvovirus*), PARV4 (*Tetraparvovirus*), AAV2 (*Dependoparvovirus*), and B19 (*Erythroparvovirus*). Radially colored capsid surface representations (blue to red) are viewed along the two-fold axis and were generated using Chimera [25]. TBD: to be determined.

This study extends the available capsid structural atlas of the subfamily *Parvovirinae* to six of the ten genera (Figure 7). The newly added structures of AMDV and PARV4 will provide and add to the structural platform for functional annotation of these viruses. Currently, the cellular receptors and many steps of the viral life cycle for these viruses are unknown, and elucidation of the capsid structures may help to understand their disease mechanisms at a molecular level.

In the case of AMDV, similar to B19 and HBoV1, antibody-dependent enhancement (ADE) of infection was described as a form of entry to host cells for this virus, complicating vaccination strategies [41,42,43,44]. The capsid structure of AMDV may help to identify the epitopes of the antibodies, as in studies on other parvoviruses [45]. Alternatively, if ADE post-vaccination cannot be prevented, the capsid structures may help in the development of therapeutics directly targeting the capsid.

## Figures and Tables

**Table 1:** Summary of cryo-EM data collection and refinement statistics for PARV4 and AMDV.

Cryo-EM Data and Refinement Parameters	PARV4	AMDV
Total number of micrographs	1324	2254
Defocus range (µm)	0.5–2.0	0.5–2.0
Electron dose (e^−^/Å^2^)	60	59
Frames/micrograph	52	102
Pixel size (Å/pixel)	0.91	0.95
Capsids used for final map	5248	93,393
Resolution of final map (Å)	3.12	2.37
**PHENIX model refinement statistics**		
Residue range	15–552	19–565
Map CC	0.846	0.889
RMSD bonds (Å)	0.02	0.01
RMSD angles (°)	1.05	0.92
All-atom clash score	17.6	9.12
**Ramachandran plot**		
Favored (%)	98.0	95.1
Allowed (%)	2.0	4.6
Outliers (%)	0	0.3
Rotamer outliers (%)	0	0.2
C-β deviations	0	0

**Table 2:** VP and loop amino acid length comparison of AMDV with other members of the *Parvovirinae*.

AMDV vs.	VP1u	Major VP	VR I	VR II	VR III	VR IV	VR V	VR VI	VR VII	VR VIII	HI loop	VR IX
CPV	−95 ^#^	+61 ^#^	+6	+1	+7	+8	0	+5	+35	−4	−2	+6
HBoV1	−86 *	+105	+14	+4	+11	+22	−25	+12	+42	+21	+2	−1
AAV2	−159 *	+114	+14	+4	+25	+13	−20	+5	+39	+9	−3	+4
PARV4	−319	+95	+15	+4	+18	+19	−16	−4	+31	+5	−2	−4
B19	−184	+93	+3	+3	+16	+21	−20	+3	+39	+13	−1	−5

^#^ Example: AMDV has 95 aa less than CPV in VP1u and 61 aa more in the major VP. * Including the VP1/2 common region.

## Data Availability

The PARV4 and AMDV cryo-EM-reconstructed density maps and models built for their capsids were deposited in the Electron Microscopy Data Bank (EMDB) with the accession numbers EMD-28522/PDB ID 8EP9 (PARV4), EMD-28514/PDB ID 8EP2 (AMDV VP1/2), and EMD-28514 (AMDV-VP2).
